# Genome-wide association and genomic prediction for iron and zinc concentration and iron bioavailability in a collection of yellow dry beans

**DOI:** 10.3389/fgene.2024.1330361

**Published:** 2024-02-06

**Authors:** Paulo Izquierdo, Rie Sadohara, Jason Wiesinger, Raymond Glahn, Carlos Urrea, Karen Cichy

**Affiliations:** ^1^ Department of Plant, Soil and Microbial Sciences, Michigan State University, East Lansing, MI, United States; ^2^ USDA-ARS, Robert W. Holley Center for Agriculture and Health, Ithaca, NY, United States; ^3^ Department of Agronomy and Horticulture, Panhandle Research and Extension Center, University of Nebraska-Lincoln, Scottsbluff, NE, United States; ^4^ USDA-ARS, Sugarbeet and Bean Research Unit, East Lansing, MI, United States

**Keywords:** biofortification, iron bioavailability, GWAS-assisted genomic prediction, *Phaseolus vulgaris* L, diversity panel

## Abstract

Dry bean is a nutrient-dense food targeted in biofortification programs to increase seed iron and zinc levels. The underlying assumption of breeding for higher mineral content is that enhanced iron and zinc levels will deliver health benefits to the consumers of these biofortified foods. This study characterized a diversity panel of 275 genotypes comprising the Yellow Bean Collection (YBC) for seed Fe and Zn concentration, Fe bioavailability (FeBio), and seed yield across 2 years in two field locations. The genetic architecture of each trait was elucidated via genome-wide association studies (GWAS) and the efficacy of genomic prediction (GP) was assessed. Moreover, 82 yellow breeding lines were evaluated for seed Fe and Zn concentrations as well as seed yield, serving as a prediction set for GP models. Large phenotypic variability was identified in all traits evaluated, and variations of up to 2.8 and 13.7-fold were observed for Fe concentration and FeBio, respectively. Prediction accuracies in the YBC ranged from a low of 0.12 for Fe concentration, to a high of 0.72 for FeBio, and an accuracy improvement of 0.03 was observed when a QTN, identified through GWAS, was used as a fixed effect for FeBio. This study provides evidence of the lack of correlation between FeBio estimated *in vitro* and Fe concentration and highlights the potential of GP in accurately predicting FeBio in yellow beans, offering a cost-effective alternative to the traditional assessment of using Caco2 cell methodologies.

## Introduction

Dry bean is the most important legume for human consumption worldwide (FAO et al., 2022) providing high levels of protein, dietary fiber, and micronutrients such as iron (Fe) and zinc (Zn) (Uebersax et al., 2022). Dry beans are also an appealing crop choice for biofortification because of their low environmental footprint, ability for symbiotic nitrogen fixation, and long shelf life that minimizes food waste (Willett et al., 2019). Biofortification initiatives since 2005 have focused on increasing dry bean Fe and Zn concentrations through breeding as a means to improve the nutritional status of humans with Fe and Zn deficiencies (Beebe, 2020). Iron concentrations in beans are projected to decrease in climate change scenarios of rising CO2 (Smith et al., 2017). Combatting climate change necessitates a shift toward plant-based diets, which, though beneficial for the environment, typically offer lower iron bioavailability compared to animal-based alternatives (van Wonderen et al., 2023). This transition is likely to exacerbate human iron deficiency issues in the future (MacDiarmid and Whybrow, 2019). Consequently, emphasizing iron and its bioavailability in bean breeding programs becomes crucial to mitigate this nutritional concern.

As bean breeders have worked for the last 2 decades to biofortify beans by increasing minerals concentrations, major efforts have been made to understand the genetic architecture of seed Fe and Zn accumulation (Cichy et al., 2009; Blair et al., 2011; Blair and Izquierdo, 2012; Blair et al., 2013; Mahajan et al., 2017; Caproni et al., 2020; Delfini et al., 2021; Gunjača et al., 2021; Cichy et al., 2022; Nazir et al., 2022). A meta-QTL (MQTL) analysis identified 12 stable MQTLs over different genetic backgrounds and environments (Izquierdo et al., 2018). While there has been a push towards breeding for increased seed micronutrient concentration, multiple studies have also underscored the value of breeding for enhanced Fe bioavailability (Katuuramu et al., 2018; Glahn et al., 2020; Katuuramu et al., 2021). These studies have indicated that Fe concentration and Fe bioavailability are not necessarily positively correlated. Increased Fe levels are often associated with increased amounts of polyphenols and phytate in the seed and both compounds form complexes with Fe that can pass through the human small intestine undigested, thereby negating the potential benefit of the higher Fe concentration (Tako et al., 2015).

Seed Fe and Zn concentrations are quantitative traits controlled by many loci across the genome in dry beans (Izquierdo et al., 2018) and are strongly influenced by the environment (Katuuramu et al., 2021). Although Fe bioavailability is also a quantitative trait, a study reported across nine locations in Uganda found it to be stable across environments (Katuuramu et al., 2021). The Caco-2 cell bioassay is the most cost-effective and practical method for assessment of Fe bioavailability from foods; however, the model requires highly trained technicians and laboratory resources that are often out of reach for most breeding programs (Glahn et al., 1998). One study in common bean has dissected the genetic architecture of Fe bioavailability through genome-wide association studies (GWAS), identifying five SNP associations distributed on chromosomes 6, 7, and 11 with phenotypic variability explained by the associated markers ranging from 8% to 13% (Katuuramu et al., 2018).

Genomic prediction uses genotype and phenotype from training datasets to estimate the phenotypes of new lines within a testing dataset, thereby reducing the need for extensive phenotyping and increasing selection intensity (Bernardo, 2020). This approach predicts phenotypes using all genetic markers collectively (Meuwissen et al., 2001), and the accuracy, often represented as the Pearson correlation of observed vs predicted phenotypes, depends on several factors. These include the heritability of the trait, linkage disequilibrium (LD) with causal loci, and the size of the population. The Genomic Best Linear Unbiased Predictor (GBLUP) is a widely employed parametric linear model that captures the additive relationships between lines. However, semiparametric models like the Reproducing Kernel Hilbert Space (RKHS) can potentially identify non-additive effects, thereby potentially enhancing prediction accuracy (Gota and Gianola, 2014; Cuevas et al., 2016). Another critical aspect of genomic prediction is the genetic relationship between the training and testing datasets (Voss-Fels et al., 2019). The composition of the training data set and its relationship with the new lines to be predicted are crucial for maximizing prediction accuracy (Isidro et al., 2021; de los Campos et al., 2013). While several approaches have been proposed to optimize the training population, most of them have the assumption that one training is optimal for all individuals in the testing dataset (Lopez-Cruz et al., 2022). Considering that a high level of genetic heterogeneity is plausible in breeding programs, using only one optimal population may include individuals distantly related to the individuals in the testing dataset, reducing predictive ability (Lorenz and Smith, 2015). Recently, a sparce selection index (SSI) was proposed to identify a training set for each individual in the testing set (Lopez-Cruz and de los Campos, 2021). The use of this approach has increased prediction ability in multigeneration data up to 10% and 17% in wheat and maize, respectively, compared to the GBLUP (Lopez-Cruz et al., 2021; Lopez-Cruz et al., 2022).

Large genetic variability exists for Fe and Zn concentration, as well as for Fe bioavailability, both among and within market classes (Katuuramu et al., 2021). This variability provides evidence that achieving genetic gain in these traits is feasible. However, due to the complex genetic architecture of these traits, the use of genomics approaches is necessary to identify causative alleles and to evaluate prediction models. These approaches are essential for enhancing genetic gain in these traits, thereby increasing the nutritional value of new cultivars. Furthermore, there is a lack of information in the literature about the performance of prediction models trained on diversity panels when implemented in breeding lines. To address these challenges, we utilized Single Nucleotide Polymorphism (SNP) markers to identify genomic regions associated with Fe and Zn concentration and Fe bioavailability in a Yellow Bean Collection (YBC). Additionally, we evaluated the prediction accuracy of genomic prediction in the YBC and investigated whether the inclusion of SNP markers as fixed effects improves prediction accuracy. Finally, we assessed the predictive ability of models trained using the YBC on advanced yellow lines, exploring the potential of using genomic prediction to enhance nutritional quality in the yellow bean market class.

## Materials and methods

### Plant material

The yellow bean collection (YBC) comprised of 275 *Phaseolus vulgaris* L. accessions was grown at the Michigan State University Montcalm Research Farm in Entrican, MI, and at University of Nebraska field sites in Scottsbluff and Mitchell, Nebraska, during 2018 and 2019 growing seasons, respectively. A detailed description of each accession in the YBC is provided by Sadohara et al. (2022). In all years and locations, the YBC was grown in a randomized complete block design with two replications. Additionally, 82 F5 yellow bean breeding lines were planted at the Montcalm Research Farm in 2019. These lines originated from biparental crosses involving seven Andean accessions, six of which are part of the YBC, as parents. These accessions were chosen for breeding due to their agronomic characteristics, stable yield across seasons, desirable seed color, short cooking times, and high iron bioavailability. The YBC planted in both years was used as the training set, while the 82 lines evaluated in 2019 were used as the prediction set to simulate the implementation of GP using a pre-breeding population. The local standard agricultural practices were followed for research plot scale dry bean production (Sadohara et al., 2022). Seed weight (SW) and yield (YD) were collected after harvest. SW (g) was obtained from weighing 100 seeds, and YD (kg ha^−1^) was calculated based on the plot size and corrected to the moisture content in the seed of 18%.

### Mineral concentrations (Michigan and Nebraska)

The protocol described by Glahn et al. (2020) was used to measure Fe and Zn concentration from raw seeds in the YBC grown in Michigan (MI) and Nebraska (NE) in 2018 and 2019 and the breeding lines grown in MI in 2019. Briefly, seeds were rinsed and cleaned with distilled water to remove dust and debris. The cleaned seeds were lyophilized and milled, and 0.50 g of the powder was predigested in boro-silicate glass tubes with concentrated ultrapure nitric acid and perchloric acid mixture (60:40 v/v) for 16 h at room temperature. Samples were then placed in a digestion block and heated incrementally over 4 h to a temperature of 120°C with refluxing. After incubating at 120°C, ultrapure nitric acid was subsequently added to each sample before raising the digestion block temperature to 145°C for an additional 2 hr. The temperature was then raised to 190°C for 10 min to evaporate remaining acid before cooling to room temperature. Digested sample was re-suspended in 20 mL of ultra-pure water before analysis using ICP-AES (inductively couple plasma atomic emission spectrometry; Thermo iCAP 6500 Series, Thermo Scientific, Cambridge, United Kingdom) with quality control standards following every 10 samples. All samples were digested and measured with 0.5 µg/mL of Yttrium (final concentration) purchased from High Purity Standards (10 M67-1) as an internal standard to ensure batch-to batch accuracy.

### Fe bioavailability (Michigan only)

The YBC grown in MI in 2018 and 2019 was cooked using an automated Mattson cooker, as reported previously (Sadohara et al., 2022). The cooked samples were lyophilized and milled, and 0.50-g of powder was subjected to an *in vitro* digestion/Caco-2 cell culture model for the determination of Fe bioavailability, as described previously by Glahn et al. (1998). Fe uptake is measured as the increase in Caco-2 cell ferritin production (ng ferritin per milligram of total cell protein) after exposure to simulated gastric and intestinal digest. Fe bioavailability is expressed as a percentage score of Caco-2 cell ferritin formation that is relative to a cooked/lyophilized/milled white kidney bean (Snowdon). The white kidney bean reference control was run with each bioassay to index the ferritin/total cell protein ratios of the Caco-2 cells over the course of multi-year experiment. The Snowdon white kidney bean was used as a reference control because this cultivar is commercially produced in North America and has high Fe bioavailability due to the lack of polyphenols that inhibit the absorption of iron (Wiesinger et al., 2019). The mean ferritin formation values of Snowdon across assays were 19.71 and 15.78 ng ferritin/mg total cell protein in 2018 and 2019, respectively. Fe bioavailability was not measured in the 82 breeding lines.

### Statistical analyses

The rows and columns from the field were used as random effects to fit a linear mixed model for SW and YD using the functions “SpATS” and “SAP” for the R package SpATS (Rodríguez-Álvarez et al., 2018). The effect of the genotype on the phenotype was fitted as fixed to obtain the best linear unbiased estimator (BLUE) of SW and YD. The mineral concentrations were collected in one field replication in Michigan and Nebraska during two growing seasons (2018–2019), while Fe bioavailability was collected in one field replication only in Michigan during two growing seasons (2018–2019). The mean of two technical replications was used as the mineral concentration and Fe bioavailability for each environment and year. The variance component analysis was conducted using the R package statgenGxE, adopting a factorial structure of locations per year (StatgenGxE, 2023). In this mixed linear model, terms for year, location and location:year were treated as fixed effects, while the effects of genotype, genotype:year, and genotype:location were treated as random.

### Genotypic data

DNA was extracted from trifoliate leaves using NucleoSpin Plant II Kit (Macherey–Nagel, Duren, Germany) following the “Genomic DNA from plants” protocol as described previously by Sadohara et al. (2022). DNA concentration was measured using Quant-iTTM PicoGreenTM dsDNA Assay Kit (Invitrogen, Waltham, MA, United States), and 10 ng/lL of DNA was used for GBS library preparation with a single restriction enzyme, ApeKI. Each plate of 96-wells was sequenced in a lane of an Illumina HiSeq platform using single-end reads. The libraries were demultiplexed using NGSEP (v3.1.2) (Tello et al., 2019). Adapters and low-quality bases from the raw sequencing data were trimmed using Cutadapt v 1.16, and the processed reads were aligned to the reference genome of *P. vulgaris* v2.1 G19833 (Schmutz et al., 2014) using Bowtie2 (v2.2.30) (Langmead and Salzberg, 2012) with default parameters. The SNP calling was carried out by using NGSEP software following the recommended parameters for GBS data (Perea et al., 2016). The merged genotypic matrix was filtered with NGSEP for variants that were in the predicted repetitive regions of the common bean genome (Lobaton et al., 2018), non-biallelic, genotype quality above 30, a maximum observed heterozygosity of 0.05 per SNP, more than 50% of missing data per site, and minor allele frequency (MAF) above 0.05. Besides, SNPs in linkage disequilibrium above 0.9 using a window of 500 SNPs were removed using Bcftools (Li, 2011). The resulting genotype matrix was imputed using Beagle V5.4 with default parameters (Browning et al., 2018).

### Genome-wide association study (GWAS)

The GWAS was conducted using the Fixed and Random Model Circulating Probability Unification (FarmCPU) method, as implemented in GAPIT v3 (Wang and Zhang, 2021). The YBC accessions from each year and location were used for this analysis. FarmCPU iteratively adjusts for fixed (individual SNP markers) and random effects (polygenic background and population structure), enhancing true association detection. A Shapiro-Wilk test was carried out on the phenotypic data, and traits not considered normal (*p*-value <0.05) were transformed using the rank-based inverse normal transformation (INT). Subsequently, GWAS was conducted on both transformed and untransformed phenotypes. Significant associations were identified using a Bonferroni-corrected threshold of *α* = 0.05.

### Genomic prediction

Four prediction models were assessed in the YBC: The Reproducing Kernel Hilbert Space (RKHS) regression, sparse selection indices (SSI), and RKHS and SSI using QTN identified through GWAS as fixed variables. The RKHS regression was implemented using the kernel averaging (KA) approach (de los Campos et al., 2010) with kernels estimated using extreme bandwidth parameters (0.2, 1, 5) (González-Camacho et al., 2012). The Gaussian kernels utilize a positive-definite kernel represented by: K = exp (-*θ* $\left.d_{ij}^{2}\right)$ , where K represents the kernel, θ is the bandwidth parameter, and $d_{ij}^{2}$ represents the scaled squared Euclidean distance between individuals *i* and *j* based on their SNPs (Lopez-Cruz et al., 2021).

The SSI was obtained by imposing an L1-penalty on a selection index using additive genomic relationships (GSSI) (Lopez-Cruz and de los Campos, 2021). To optimize the penalization value λ in SSI, 10-fold cross validation was carried out in the training subset, and SSI was derived over a grid of 100 values of *λ*. The accuracy measured as the Pearson correlation between SSI and each trait was used to identify the value of *λ* that maximized accuracy in each cross validation. The optimal value of *λ* was defined as the average value of *λ* that maximized accuracy across each cross-validation and was used to predict the validation subset. The training-validation procedure described above for KA and SSI was repeated 100 times by randomly assigning samples from the training set into training and validation subsets and was implemented using the R packages SFSI and BGLR (Lopez-Cruz et al., 2020; Pérez-Rodríguez and de Los Campos, 2022). The prediction ability for all models is expressed as a Pearson correlation coefficient between the observed and predicted values in the validation subset.

### Prediction set

To simulate the implementation of GP, 82 breeding lines from 2019 were included as the prediction set, as previously described. The YBC was employed to train the KA and SSI models. Given that the breeding lines are derived from crosses between Andean accessions, the models were trained with two distinct YBC datasets: one encompassing all accessions and another restricted to Andean accessions. Additionally, to train the models, different proportions of individuals of each bi-parental family (0%, 10%, 20%, 30%) were randomly assigned to the training sets. The procedure described above was repeated 100 times and was implemented using the R packages SFSI and BGLR (Lopez-Cruz et al., 2020; Pérez-Rodríguez and de Los Campos, 2022).

## Results

### Phenotypic data

The YBC was grown in field locations in MI and NE in 2018 and 2019 and was evaluated for seed yield, seed weight, Fe and Zn concentration in MI and NE 2018–2019, and FeBio was evaluated in Michigan 2018–2019. The YBC exhibits large variability for agronomic and nutritional quality traits (Table 1 and Supplementary Figures S1, S2). The FeBio distribution was bimodal in the two growing seasons measured in Michigan. The concentration of Fe ranged from 43 to 118 μg/g in Michigan 2018, 39–106 μg/g in Michigan 2019, 28–76 μg/g in Nebraska 2018, and 36–72 μg/g in Nebraska 2019 (Table 1). Four genotypes had concentrations above 100 μg/g in Michigan, three in 2018 (YBC050, YBC136, and YBC279) and one in 2019 (YBC171). The concentration of Zn ranged from 20 to 41 μg/g in Michigan 2018, 17–41 μg/g in Michigan 2019, 19–37 μg/g in Nebraska 2018, and 21–43 μg/g in Nebraska 2019 (Table 1). FeBio values in Michigan varied from 11% to 151% in 2018 and from 13% to 118% in 2019, compared to the reference control Snowdon (Table 1). Twenty-seven genotypes had higher FeBio values than the high FeBio check variety, Snowdon, in 2018 and 2019.

**TABLE 1:** The mean and the range of yield, seed weight, Fe bioavailability, Fe and Zn concentration of the YBC grown in MI and NE in 2018 and 2019.

Trait	MI 2018		MI 2019		NE 2018		NE 2019	
	Mean (SD)	Range	Mean (SD)	Range	Mean (SD)	Range	Mean (SD)	Range
Yield (kg/ha)	2111 (394)	1171–3299	1649 (477)	536–3065	1974 (647)	653–5,275	686 (372)	52–2124
SW (g)	38 (8)	18–60	40 (10)	18–73	35 (7)	15–54	30 (1)	26–35
Fe (μg/g)	69 (12)	43–118	65 (9)	39–106	45 (9)	28–76	51 (6)	36–72
Zn (μg/g)	29 (4)	20–41	28 (4)	17–41	25 (3)	18–37	27 (3)	21–43
FeBio (% of control)	54 (31)	11–151	51 (30)	13–118	-	-	-	-

The variance component analysis revealed a significant effect of genotype on the agronomic and nutritional quality traits assessed in the current study (Supplementary Table S1). The most important source of variation for Fe concentration was the location followed by the Year:Location, while genotype explained most of the variation in Zn concentration and FeBio. Indeed, the genotype explained 93%, 5%, and 37% of the phenotypic variation in FeBio, Fe, and Zn concentration, respectively. The large effect of the genotype on the variability of FeBio is supported by the high h^2^ of FeBio found in 2018 (0.85) and 2019 (0.91).

**FIGURE 1:**
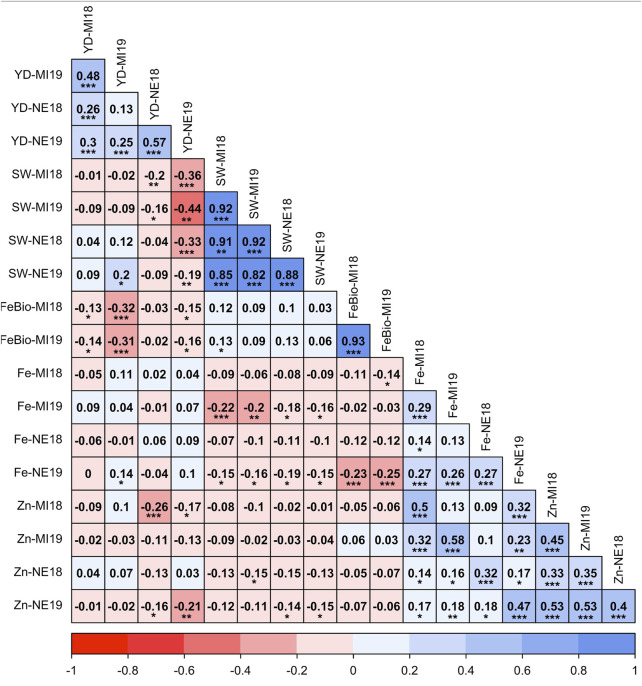
Pearson’s correlation coefficients between agronomic traits (Seed weight (SW), yield (YD), mineral concentration (Fe, Zn), and Fe bioavailability (FeBio). Significance of correlations indicated as ***: *p* < 0.001; **: *p* < 0.01; *: *p* < 0.05.

Despite the differences between location and years, the measurements of all traits were positively correlated across years (Figure 1). The correlation between Fe concentration and yield was not significant in any environment, ranging from −0.05 in Michigan in 2018 to 0.10 in Nebraska in 2019, while the correlation between yield and Zn concentration was negative in both locations and years. Fe and Zn concentrations were positively correlated, ranging from r = 0.32 (*p* < 0.001) in Nebraska in 2018 to r = 0.58 (*p* < 0.001) in Michigan in 2019. Both Fe and Zn concentrations were negatively correlated with seed weight in both locations and years. The most stable trait across years (2018–2019) was FeBio, with a correlation between years of 0.93 (*p* < 0.001) in Michigan. FeBio was negatively correlated with yield in 2018 (r = −0.13, *p* = 0.05) and 2019 (r = −0.31, *p* = 0.001).

### Population structure

**FIGURE 2:**
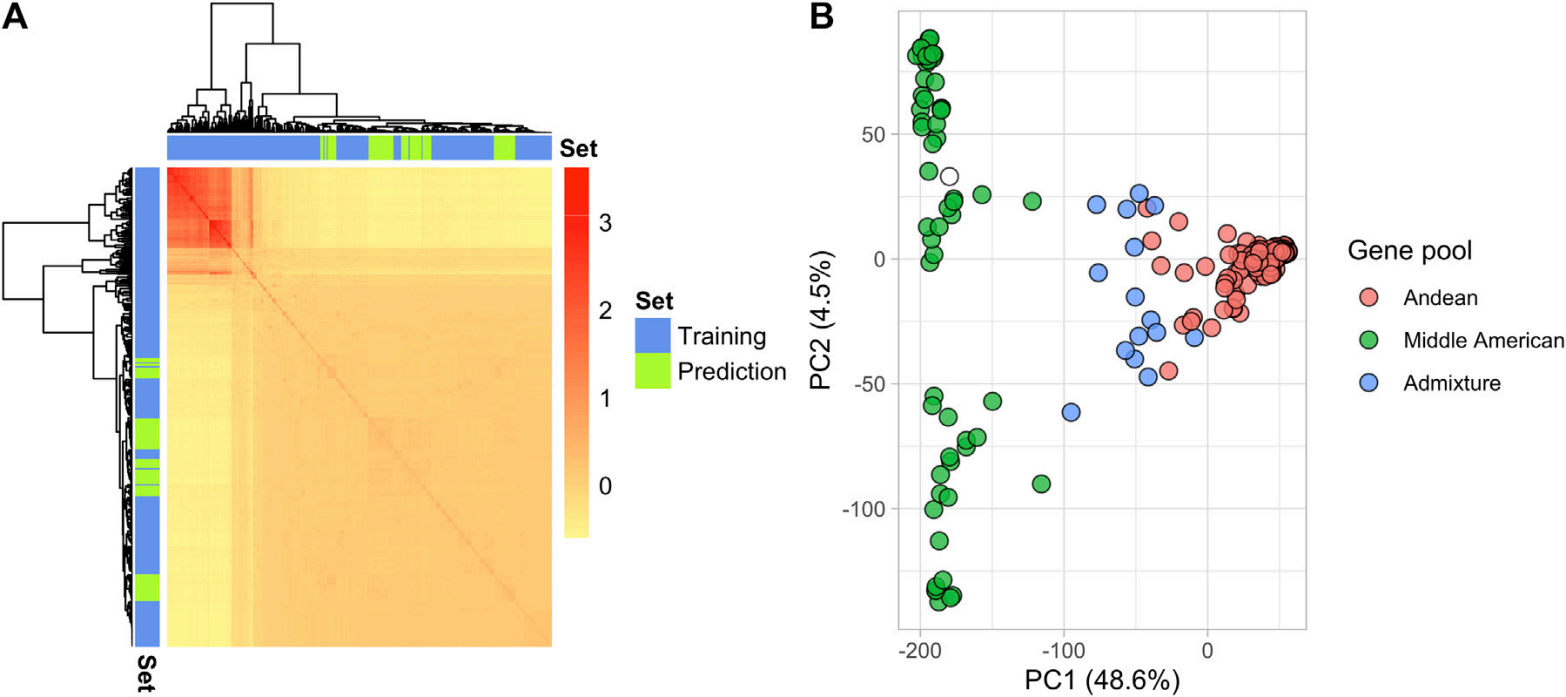
Genetic structure of the YBC and advanced yellow breeding lines with 18,357 SNPs. **(A)** Heatmap of the genomic relationship matrix. The blue and green color represent the genotypes in the training and prediction sets, respectively. **(B)** The principal component analysis shows the location of each genotype defined by the eigenvectors of the first and second principal components. The color represents the gene pool determined by Sadohara et al. (2022).

In total, 18,357 SNP markers were retained after filtering in the 275 YBC and 82 breeding lines. The genomic relation matrix revealed two groups with strong genetic relationships, representing the germplasm from the two gene pools in *P. vulgaris* (Figure 2A). As expected, the breeding lines included in 2019 were clustered in the same gene pool as their founders (Andean) (Figure 2A). Genetic diversity was evaluated through principal component analysis (PCA). The first two principal components explained 53.1% of the variance (Figure 2B). PC1 separated the Andean and Middle American gene pools as reported by Sadohara et al. (2022). However, the variance explained by PC1 (48.6%) in this study differs from the PC1 (63.8%) reported by Sadohara et al. This difference is due to different filters applied to the genotype matrix and the addition of the 82 breeding lines in the present study. Additionally, a Principal Component Analysis (PCA) conducted on the Andean accessions revealed that the parents used in the biparental crosses are closely related genetically (Supplementary Figure S3).

### GWAS

**FIGURE 3:**
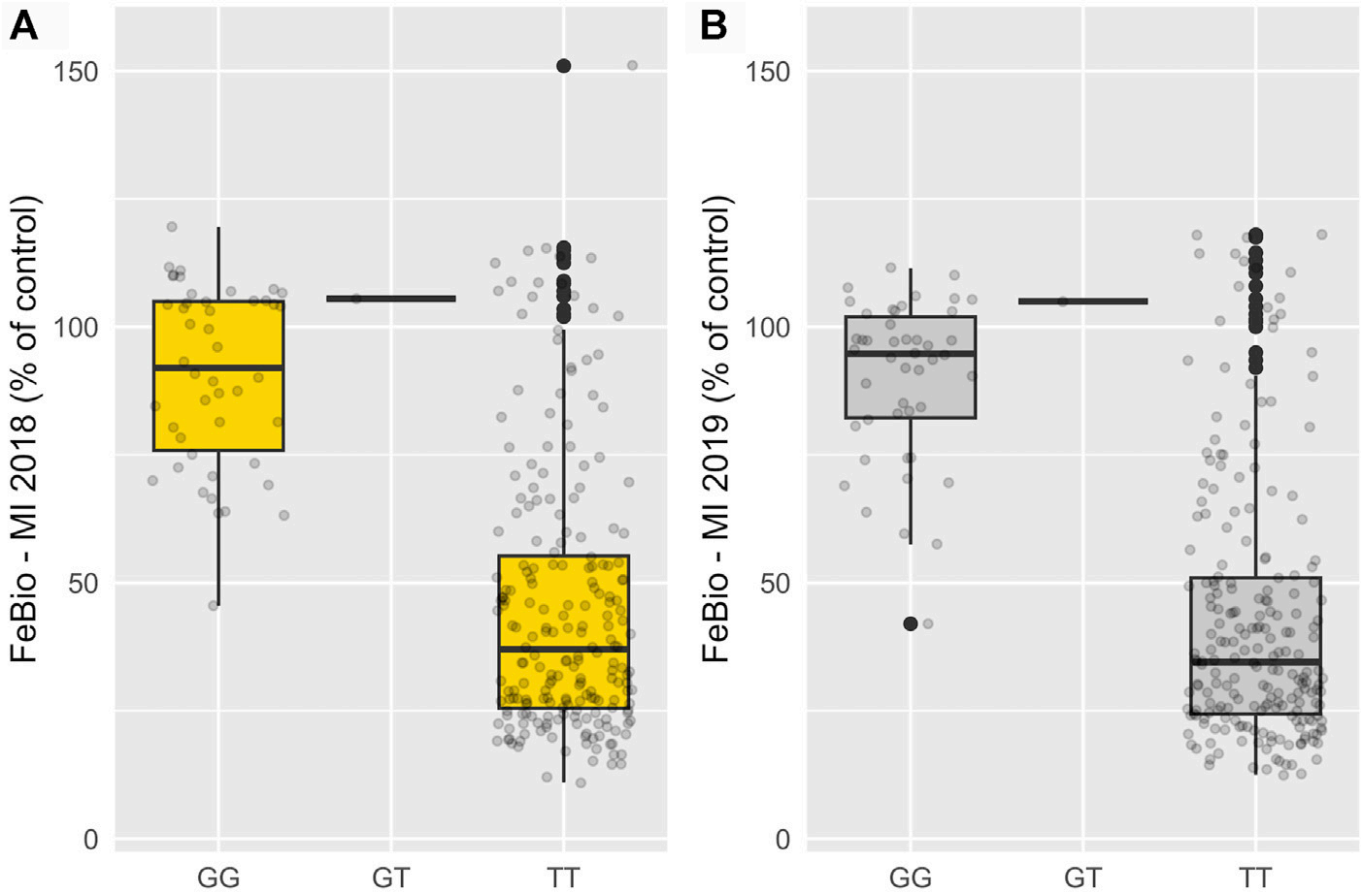
Phenotypic effect of SNP at chromosome 7 (29,2 Mb) associated with FeBio in 2018 **(A)** and 2019 **(B)**.

Marker-trait associations were evaluated using the multi-marker approach, FarmCPU. In total, 24 SNP associations were identified in the untransformed phenotypic data, and 14 in the INT data, all with significance surpassing the threshold established by the Bonferroni correction (2.72 × 10^−6^) (Supplementary Table S2). Three genomic regions on chromosomes 7, 10, and 11 were consistently associated with FeBio in Michigan across years using the untransformed phenotypes (Supplementary Figure S4). However, when analyzing the normalized, transformed phenotypes, these genomic regions on chromosomes 7, 10, and 11 showed associations in at least 1 year. In contrast, no consistent associations were identified for Fe and Zn concentrations or yield between years or locations. Notably, the association with FeBio on chromosome 7 exhibited the highest SNP effect, represented as the difference between homozygous genotypes, over the two evaluated years. (Figure 3; Table 2).

**TABLE 2:** QTN for FeBio identified by genome-wide association analysis over years using FarmCPU model.

Chr	Position	*p*-value	Year	SNP effect
7	29,169,848	4.43E-07	2018	45.2
7	29,169,848	1.75E-08	2019	46.9
10	42,422,135	5.90E-11	2018	3.3
10	42,422,135	2.67E-17	2019	3.9
11	3,626,904	4.75E-08	2018	9.3
11	3,626,904	6.77E-08	2019	8.0

### Genomic prediction–training set

**FIGURE 4:**
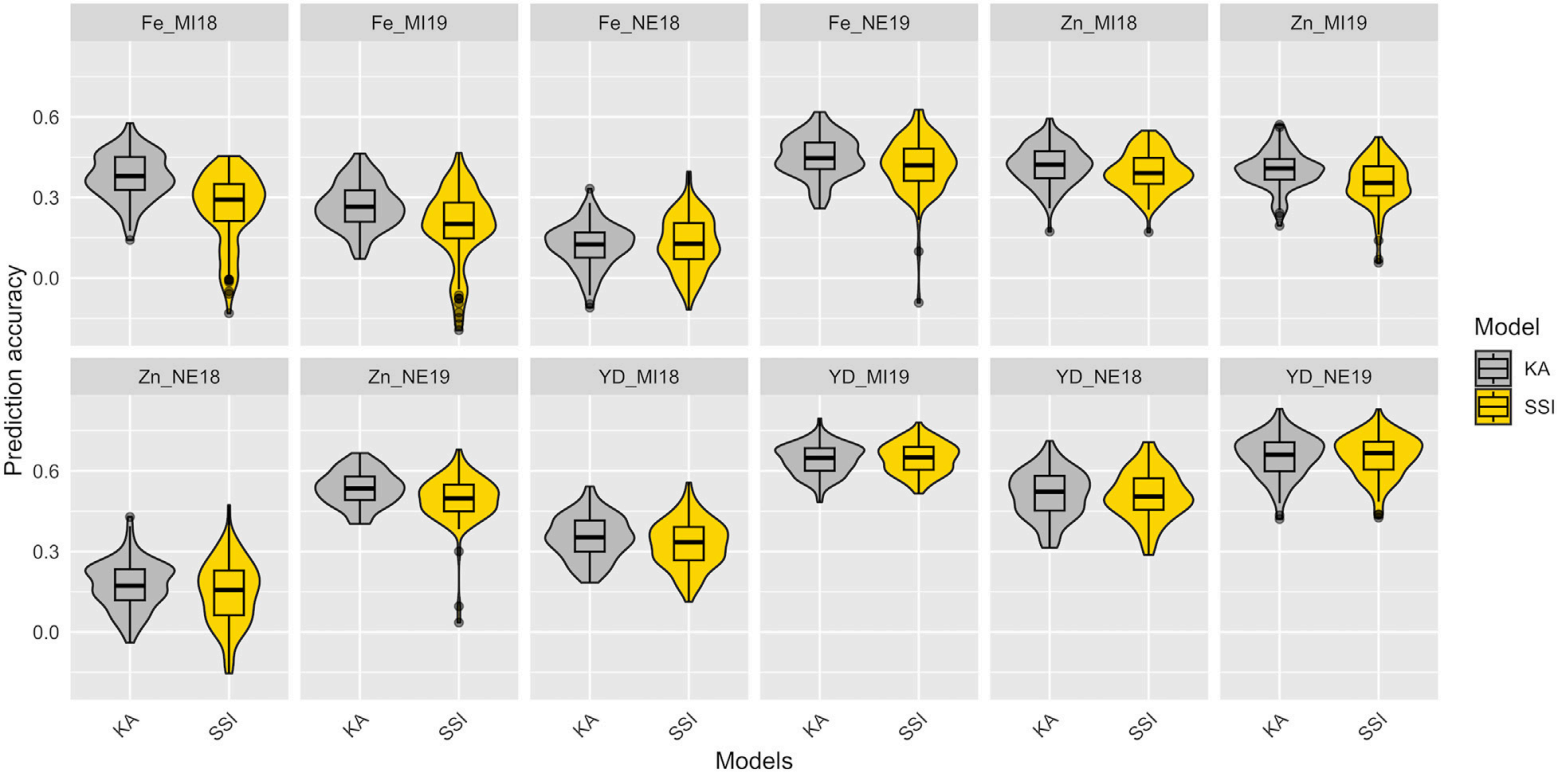
Prediction accuracy in the training data set of Fe-Zn concentration and yield using KA and SSI. The distribution of boxplots represents 100-fold cross-validations in the training set.

In total, 100 partitions were used to assess the prediction accuracy for the KA and SSI models in the training set composed of 275 YBC. The prediction accuracies of KA and SSI models for Fe and Zn concentrations and seed yield are presented in Figure 4 and Supplementary Table S3. The prediction accuracy was affected by trait heritability, and no significant differences were observed across models. KA models used all the genotypes in the training subset to train the model, while SSI used a penalization to select the individuals in the training subset to maximize the prediction ability. The average number across locations and years of individuals that support predictions using the SSI ranged from 34% to 95% of individuals in the training subset from Fe concentration and yield, respectively (Supplementary Table S3). The lowest prediction accuracy found in this study was detected in Fe concentration in Nebraska in 2018. Soil analysis revealed a pH of 8.0 at this location. The h^2^ (0.05, 0.20) and prediction ability <0.17 for Fe and Zn concentration were likely affected by the alkaline soil of this location which causes reduced iron availability for plant uptake. Average prediction accuracy in 2018 and 2019 for yield ranged from 0.33 to 0.65 in Michigan and 0.51 to 0.65 in Nebraska, respectively (Figure 4).

**FIGURE 5:**
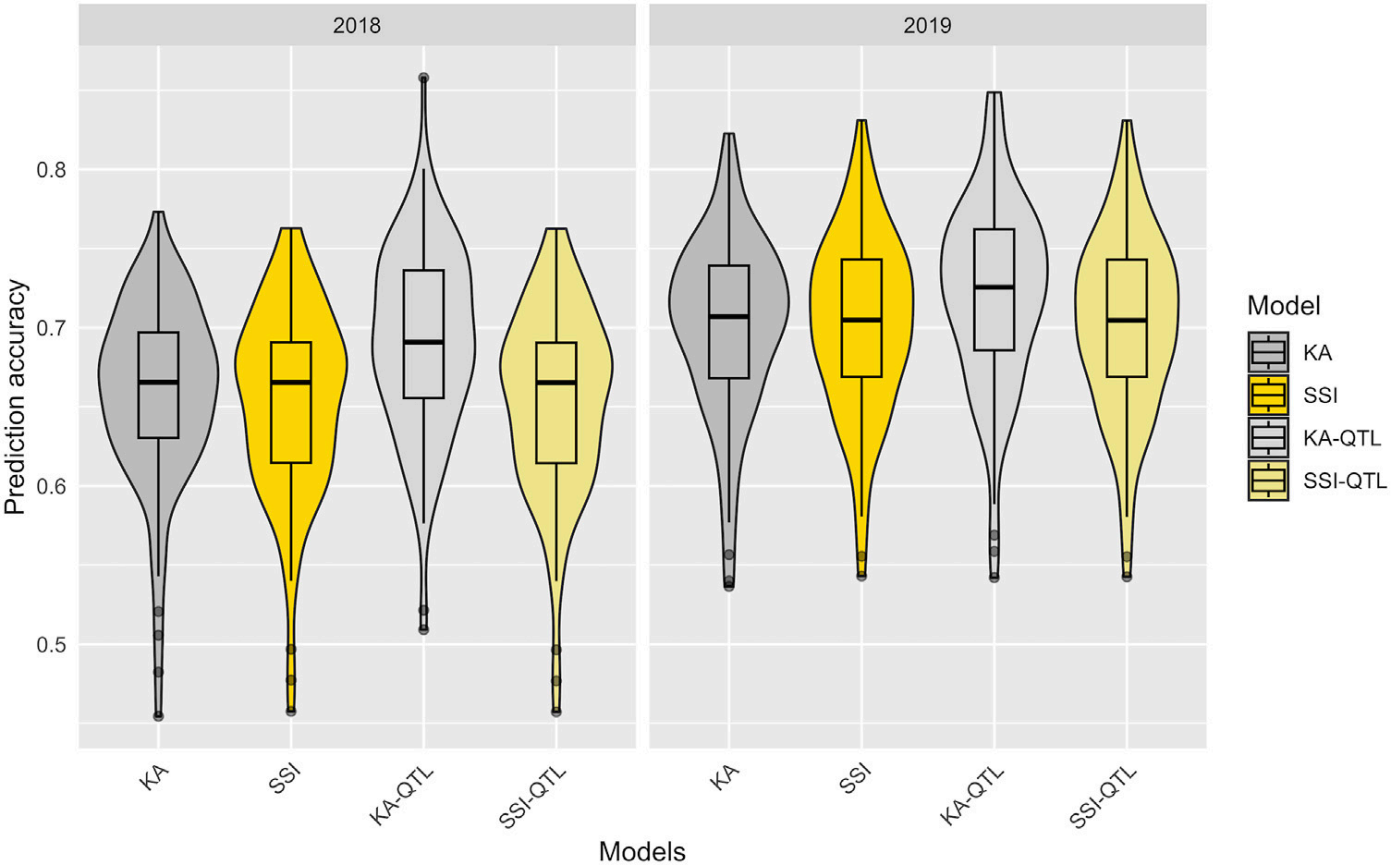
Prediction accuracy in the training data set of FeBio using KA, SSI, and QTN as fixed effect. The distribution of boxplots represents 100-fold cross-validations in the training set.

Utilizing the 100 partitions previously described, the prediction accuracy of both the KA and SSI models was evaluated for FeBio prediction by incorporating the major QTN identified on chromosome 7 (Table 1). While there was no discernible difference in prediction accuracy between the KA and SSI models without QTN information, the inclusion of the major QTN enhanced the predictive capability of the KA model. Conversely, the SSI model’s prediction accuracy remained unchanged with the addition of the QTN (Figure 5, Supplementary Table S3).

### Genomic prediction–prediction set

**FIGURE 6:**
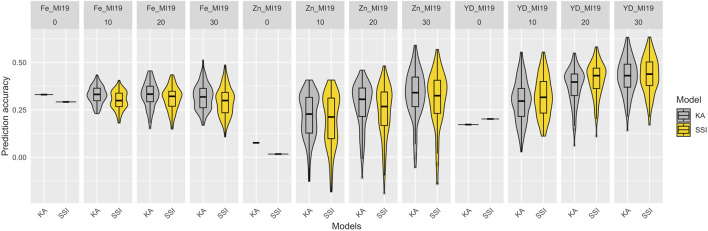
Prediction accuracy of Fe and Zn concentration and seed yield (YD) in the 2019 prediction set comprised of 82 additional breeding lines. The models were trained using the entire Yellow Bean Collection (YBC) and varying proportions of the prediction set (0%, 10% = 8, 20% = 16, 30% = 25) to train the models. The distribution of boxplots represents 100-fold cross-validations.

To simulate the implementation of GP with a diversity panel where some lines were used for breeding, a set of 82 breeding lines was grown in Michigan in 2019 and used as a prediction set. The prediction accuracy of KA and SSI models, which used all the YBC accessions and included varying proportions (from 0% to 30%) of the prediction set for model training is presented in Figure 6 and Supplementary Table S4. For the traits measured in the prediction set (seed yield and Fe and Zn concentrations), the prediction accuracy of Fe concentration was not improved when breeding lines were added to train the model. However, the accuracy tended to increase for seed yield and Zn concentration by adding genotypes belonging to the same families in the prediction set. Additionally, the SSI model showed lower accuracy compared to KA for Fe and Zn concentration, and SSI resulted in higher predictions for seed yield (Figure 6). Although the prediction accuracy obtained using SSI was overall lower compared with KA, the prediction is provided by, on average, <21% of the individuals used to train the model for Fe and Zn concentrations. For yield, the prediction is provided by, on average, <83% of the individuals used to train the model (Supplementary Table S4). Similar results were observed when only accessions from the Andean gene pool were used to train the models (Supplementary Table and Figure 5). However, prediction accuracy was consistently lower when the training population was reduced through the exclusion of non-Andean accessions.

## Discussion

Biofortification is an important goal included in many breeding programs of dry beans. Three assumptions are used to release biofortified cultivars worldwide: *i*) Fe concentration is stable across environments, *ii*) the average Fe concentration in nonbiofortified dry beans is ∼50 μg/g, *iii*) Fe bioavailability is positively correlated with Fe concentration (Glahn and Noh, 2021). In this study, we found that none of these assumptions are valid in the YBC. Although Fe concentration showed a positive correlation within and between locations in the two growing seasons, those correlations were < *r* = 0.3. In both years, beans planted in Nebraska showed a lower Fe concentration (∼48 μg/g) compared to Michigan (∼67 μg/g), and the reasons for this difference may be attributed to the alkaline soil (pH = 8) found in the Nebraska location used in this study. Values of pH greater than 7 have been related to iron chlorosis and zinc deficiency in plants (Westfall and Bauder, 2011). Additionally, the Fe and Zn in soil available for plant uptake is related to the concentration of these microelements in seeds (Katuuramu et al., 2021). The complex genetic architecture of Fe and Zn concentration was described in a meta-QTL analysis in seven populations of dry beans, and candidate genes related to the process of uptake, transport, and accumulation of these minerals were identified (Izquierdo et al., 2018). Due to the interactions with environmental factors and the complex genetic architecture of Fe and Zn concentrations, achieving a stable concentration of these microelements across environments presents challenges. Our results found no correlation between Fe concentration and *in vitro* Fe bioavailability and similar results have been reported in dry beans (Katuuramu et al., 2018; Glahn et al., 2020; Katuuramu et al., 2021), which suggests that increases in the Fe concentration do not translate to higher FeBio in yellow beans. For this absence of relationship, Glahn & Noh (Glahn and Noh, 2021) proposed to change the focus of biofortification from Fe concentration to FeBio in dry beans. There are two big advantages of breeding for FeBio over Fe concentration, higher heritability and delivery of absorbable Fe. We observed that FeBio was stable across years (r = 0.93), with a heritability >0.85, which was 2-fold the heritability of Fe concentration presented for both growing seasons in Michigan.

The high phenotypic variability and heritability of FeBio in this study suggest that genetic improvement is feasible. However, we observed a strong relationship between seed type and FeBio, which could limit the genetic gain of FeBio in some market classes (Supplementary Figure S6). The seed types Manteca, Mayocoba, and white showed high FeBio values, and we consider that these three market classes should be used as priority in programs seeking to identify and develop varieties that deliver more bioavailable Fe. Interestingly, using data reported by Sadohara et al. (2021), we identified that lighter color seeds from the YBC tend to have shorter cooking times. Fast-cooking time has been associated with high FeBio and with more retention of nutrients in dry beans (Wiesinger et al., 2016), which means that the use of Manteca, Mayocoba, and white seed types have the potential to yield high FeBio and nutrients in fast-cooking genotypes, traits that are appealing for nutrition and customer acceptance.

### GWAS

A strong environment effect was observed for yield, and Fe and Zn concentrations; and no consistent associations were identified for these traits on the YBC. The complex architecture of these traits and the GxE interaction has yielded hundreds of marker-trait associations across locations and populations (Izquierdo et al., 2018; Izquierdo et al., 2023), and the usage of strategies such as marker-assisted selection or GWAS-assisted genomic prediction does not appear promising for these traits (Keller et al., 2020).

FeBio exhibited large variability across different years and among individuals, with genetics playing a significant role in this variation. The bimodal distribution observed in FeBio likely stems from the trait governed by major QTNs. A particular SNP on chromosome 7 at 29.2 Mb is associated with features such as light-colored seed, hilum ring, corona, and resistance to darkening in the YBC (Sadohara et al., 2021). This SNP is proximal to the P gene (*Phvul.007G171333*) at 28.8 Mb, a transcription factor essential for flavonoid biosynthesis. This gene’s dominant allele, P, is necessary for seed coat color expression, while its recessive genotypes result in a white seed coat (McClean et al., 2018). This region on chromosome 7 was previously associated with FeBio in the Andean Diversity panel (Katuuramu et al., 2018). Furthermore, dark-colored bean seeds are observed to exhibit lower FeBio values (Wiesinger et al., 2018).

This finding further substantiates the relationship between seed color and FeBio. While the precise nature of the connection between seed color and FeBio remains elusive, several factors might explain it. Firstly, darker seeds typically have thicker seed coats. From ∼6 to 39% of seed iron in dry beans is found in seed coats (Moraghan, 2004), a tissue known to contain high levels of antinutrients that can bind to and chelate iron (Ariza-Nieto et al., 2007). Given that *i*) the SNP effect on chromosome 7 (Table 2) was close to the population mean for FeBio in both years (Tables 1, 2), *ii*) the association was consistent across years, and *iii*) the association with FeBio has been reported in other genetic backgrounds, such as the Andean Diversity panel, this region might be indicative of a major QTN for FeBio.

### Genomic prediction

In this study, we considered KA and SSI models to assess the accuracy of genomic prediction in the YBC. Across all traits, we found consistent results between the models, with prediction variations correlating with the heritability of each trait. While both models yielded comparable outcomes for the YBC, the specific individuals supporting the prediction of each line in the testing set differed. KA models used in each partition all the genotypes in the training subset, while SSI selected a particular set of support points as the optimal training set for each genotype in the testing set. The average number of supporting points selected by the SSI model was related to the complexity of each trait, ranging from 34% (FeBio) to 95% (yield).

In a multi-generational wheat dataset spanning 8 years, the SSI model demonstrated superior prediction accuracy compared to GBLUP (Lopez-Cruz et al., 2022). As Lopez-Cruz and de los Campos (Lopez-Cruz and de los Campos, 2021) highlighted, SSI tends to achieve higher accuracies than GBLUP, especially in larger datasets. Nevertheless, the margin of increased prediction accuracy of SSI over GBLUP is typically slight (less than 0.05) and is strongly influenced by both the diversity of the dataset and the size of the training set. Although the YBC represents a diverse population that might benefit from SSI, its training set consists of fewer than 185 individuals. This is in contrast to the thousands of individuals used in the training models by Lopez-Cruz and de los Campos (2021) and Lopez-Cruz et al. (2022).

To assess the implementation of genomic prediction using the YBC and a subset of the Andean accession from the YBC as training populations, 82 breeding lines were used as the prediction dataset. By adding different percentages of each breeding line from the same family, prediction accuracy increased for seed yield and Zn. Moreover, SSI demonstrated slightly better performance than KA in predicting seed yield. The better performance of SSI compared to KA in seed yield could be the result of differences in allele frequencies and LD patterns in the families to be predicted compared to the YBC. Differences in allele frequencies and LD may lead to suboptimal estimation of breeding values using all sets of individuals to train the model (Lopez-Cruz et al., 2022). Overall, prediction accuracies were slightly lower when non-Andean accessions were excluded from the training dataset. This reduction in prediction accuracy could be attributed to the smaller size of the training population and suggests that the inclusion of Middle American accessions contribute to increased prediction accuracy in the prediction dataset.

The traits studied here exhibited polygenic inheritance, as evidenced by the continuous variation observed across locations and years. However, incorporating a major QTN associated with FeBio as a fixed variable in the KA model improved accuracy by an average of 0.03 (2018) and 0.02 (2019) compared to the model without fixed effects. It is worth noting that the improved predictive ability from the fixed marker in this study is likely related to the diversity in seed colors present in the YBC. Its positive impact might diminish in germplasm with less varied seed types. However, even without the inclusion of the fixed marker, the prediction accuracy for FeBio remained high (>0.65) using both KA and SSI models, suggesting the potential of genomic prediction for this trait in dry beans.

## Conclusion

This study underscores the need to shift from the traditional biofortification strategy of merely increasing Fe concentration, advocating for developing dry bean cultivars with greater Fe bioavailability. The lack of correlation between Fe concentration and Fe bioavailability in this study aligns with findings from studies conducted in Africa and the United States (Katuuramu et al., 2018; Katuuramu et al., 2021), suggesting that reallocating resources in breeding programs centered on biofortification would be beneficial. We discovered a robust association between Fe bioavailability and seed coat color, pinpointing that lighter seed colors like Manteca, Mayocoba, and White often exhibit higher Fe bioavailability. These should be prioritized to bolster the supply of bioavailable Fe for human consumption. While Fe bioavailability’s significance is undeniable, its measurement might be out of reach for many breeding programs, and would thus require collaboration with laboratories capable of doing so. Our results indicated that genomic prediction offers high accuracy for Fe bioavailability, presenting a potential solution to mitigate the expenses and extensive duration associated with its measurement.

## Data Availability

The raw sequencing data for the dry bean individuals are available at NCBI under the accession number PRJNA1061170 (https://www.ncbi.nlm.nih.gov/bioproject/1061170). The GBS barcodes, along with the phenotype and genotype data for this study, can be found at https://github.com/pauloizquierdo/GWASAssistedGenomicPrediction-YBC.git.

## Supplementary material

SUPPLEMENTARY FIGURE S1 Bean plots of Fe, Zn in MI and Ne, and Fe bioavailability in MI. The trials were carried out in 2018 and 2019 in MI and NE. Micronutrient traits were collected in one field replication, and two technical replications were done per sample. The mean of the two technical replications was used as a measure for each environment and year.

SUPPLEMENTARY FIGURE S2 Bean plots of yield (YD) and seed weight (SW) of the yellow panel. The trials were carried out in 2018 and 2019 in MI and NE. Best linear unbiased estimator (BLUEs) were obtained for each trait and trial, adjusting for spatial effects in the field.

SUPPLEMENTARY FIGURE S3 Principal Component Analysis of Andean Accessions from the YBC and Advanced Yellow Breeding Lines Using 18,357 SNPs. The corols represent the six parental YBC Accessions in Biparental Crosses.

SUPPLEMENTARY FIGURE S4 Genome-wide association analysis of Fe bioavailability (FeBio). The red line indicates the Bonferroni threshold at α = 0.05. The black dashed lines highlight SNP-associated across years.

SUPPLEMENTARY FIGURE S5 Prediction accuracy of Fe and Zn concentration and seed yield (YD) in the 2019 prediction set comprised of 82 additional breeding lines. The models were trained using the Andean accession from the Yellow Bean Collection (YBC) and varying proportions of the prediction set (0%, 10% = 8, 20% = 16, 30% = 25) to train the models. The distribution of boxplots represents 100-fold cross-validations.

SUPPLEMENTARY FIGURE S6 Boxplots of Fe-Zn concentration and Fe bioavailability (FeBio) in the nine major seed types in the Yellow Bean Collection (YBC) in MI.

SUPPLEMENTARY FIGURE S7 Box plots of Fe, Zn, yield and seed weight (SW) of the yellow breeding lines categorized by family, evaluated in MI 2019.

SUPPLEMENTARY TABLE S1 Analysis of variance.

SUPPLEMENTARY TABLE S2 QTN identified by genome-wide association analysis using the FarmCPU model with a Bonferroni correction.

SUPPLEMENTARY TABLE S3 Prediction accuracy for micronutrient density and seed yield in the YBC using RKHS and sparse selection indices (SSI).

SUPPLEMENTARY TABLE S4 Prediction accuracy for Fe-Zn concentration and seed yield in the prediction set of 82 yellow breeding lines and the YBC using the RKHS and sparce selection indices (SSI).

SUPPLEMENTARY TABLE S5 Prediction accuracy for Fe-Zn concentration and seed yield in the prediction set of 82 yellow breeding lines and the Andean accession of the YBC using RKHS and sparce selection indices (SSI).

SUPPLEMENTARY TABLE S6 Shapiro-Wilk test results for phenotypic data in the Yellow Bean Collection.
