# Supplementary figures and images for: Genome-wide association and genomic prediction for iron and zinc concentration and iron bioavailability in a collection of yellow dry beans
Source: Front Genet. 2024 Feb 6;15:1330361. doi: 10.3389/fgene.2024.1330361 (PMC10876999; doi:10.3389/fgene.2024.1330361)

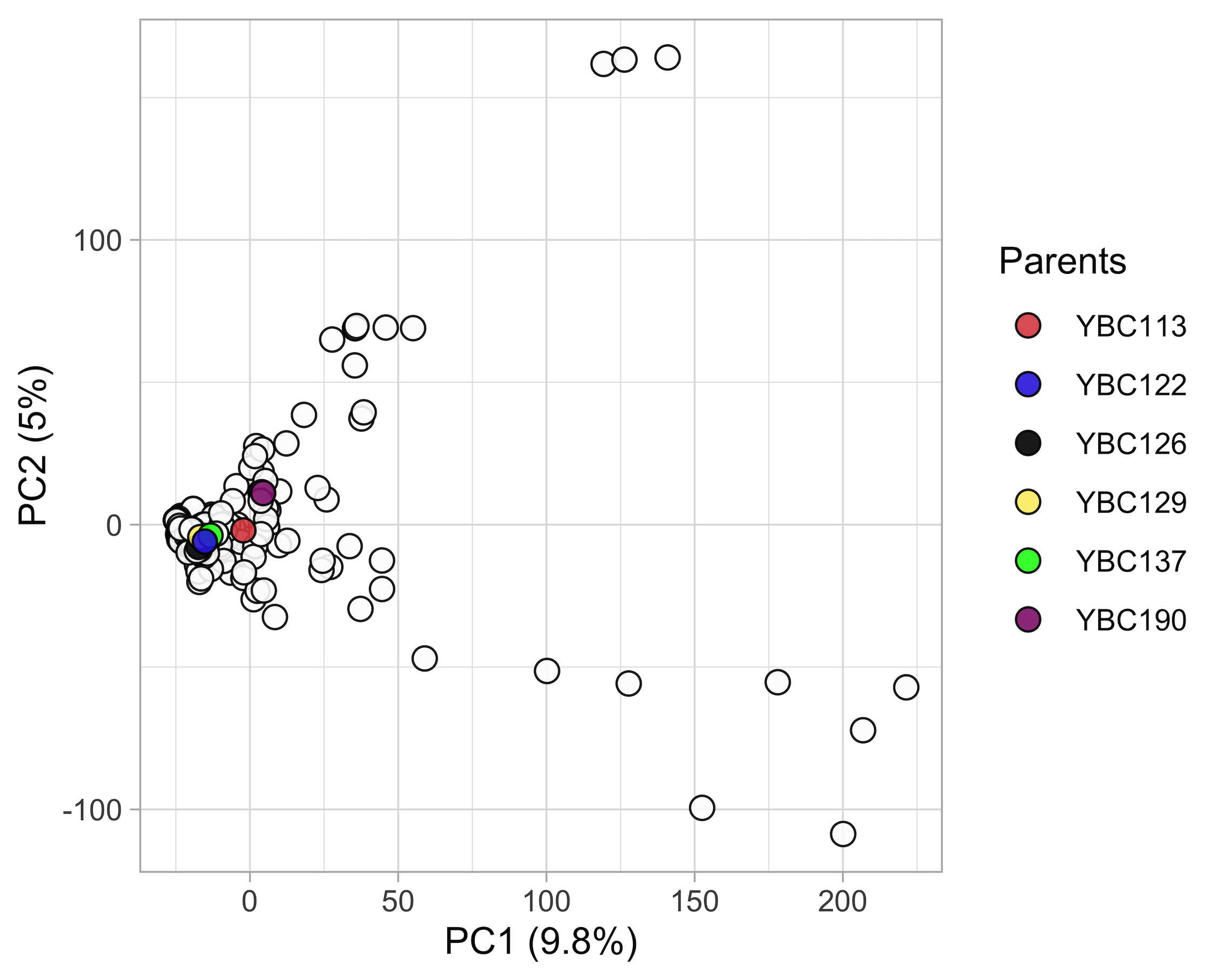

Supplement: Supplementary file 1 [file Image3.TIFF]

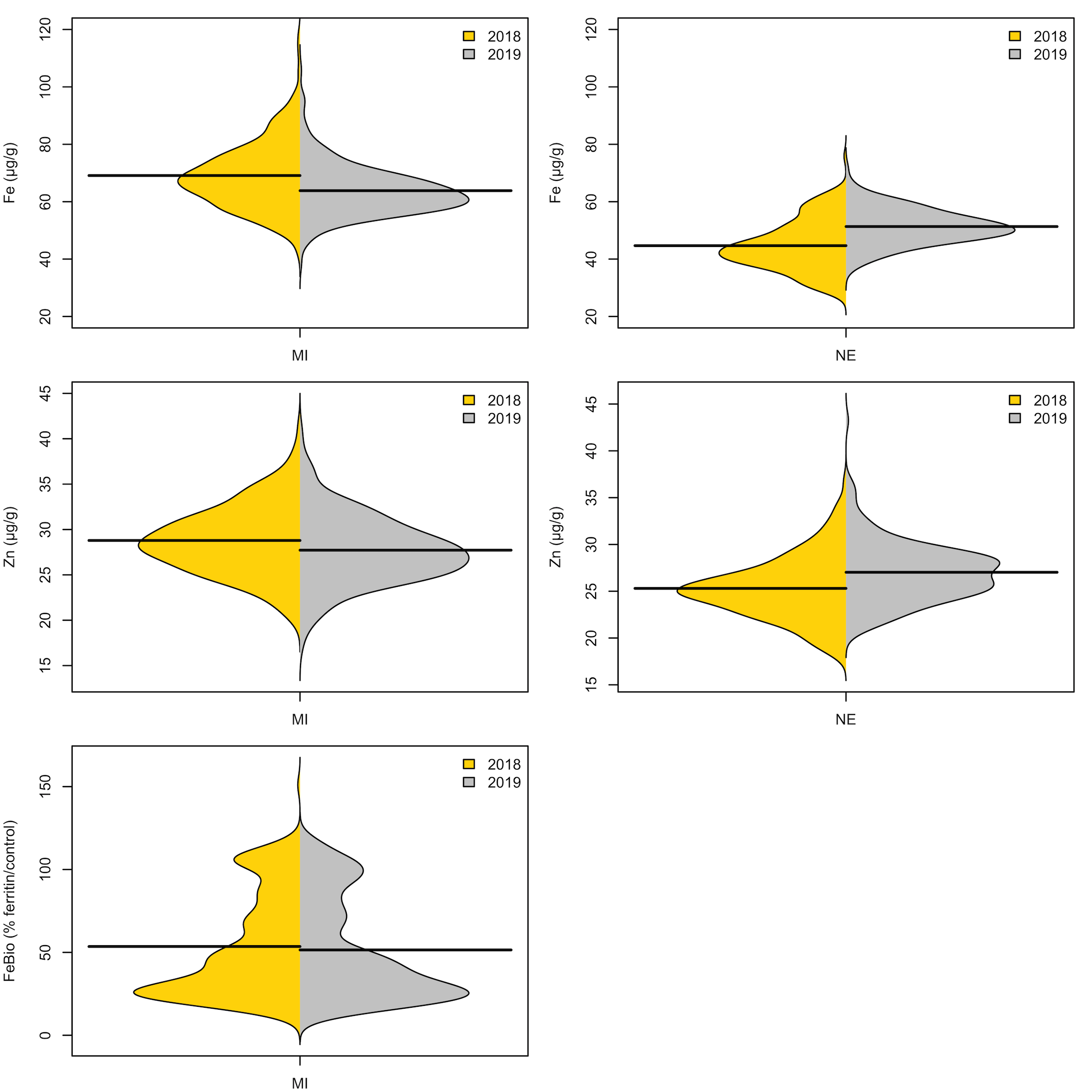

Supplement: Supplementary file 2 [file Image1.TIFF]

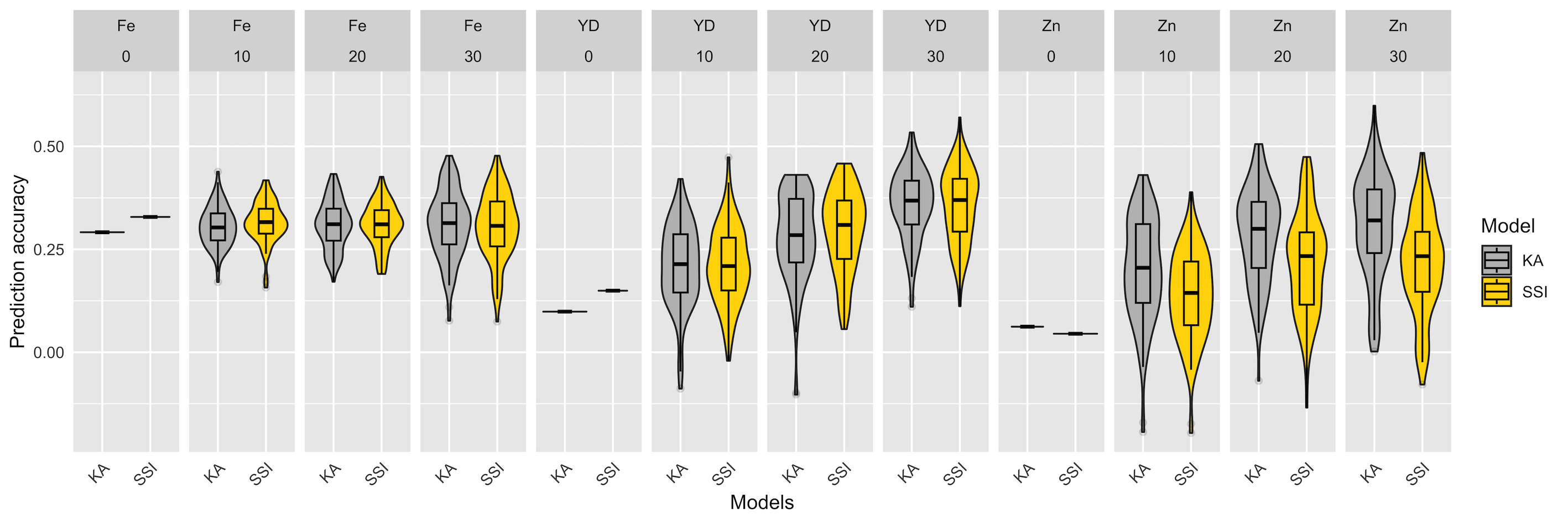

Supplement: Supplementary file 3 [file Image5.TIFF]

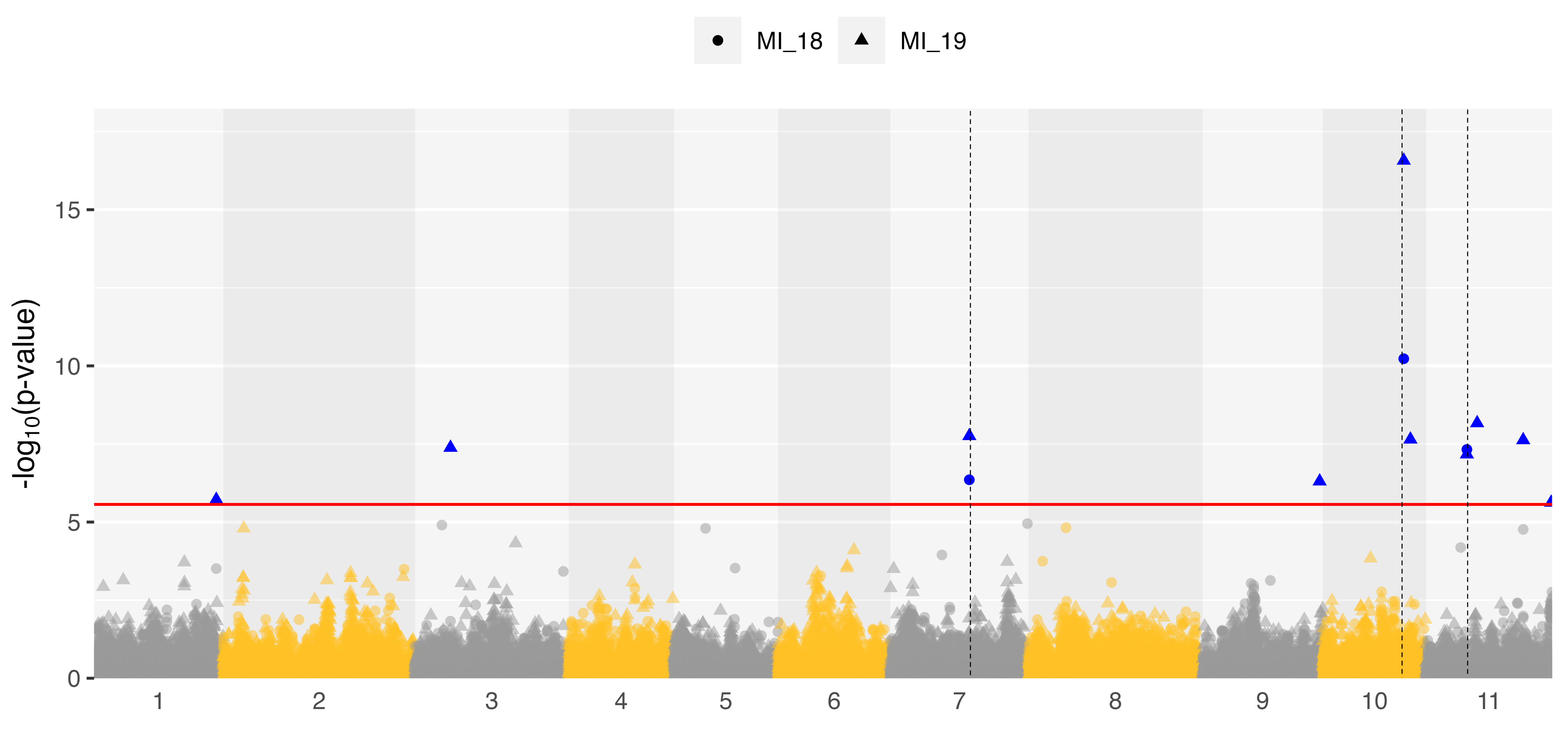

Supplement: Supplementary file 4 [file Image4.PNG]

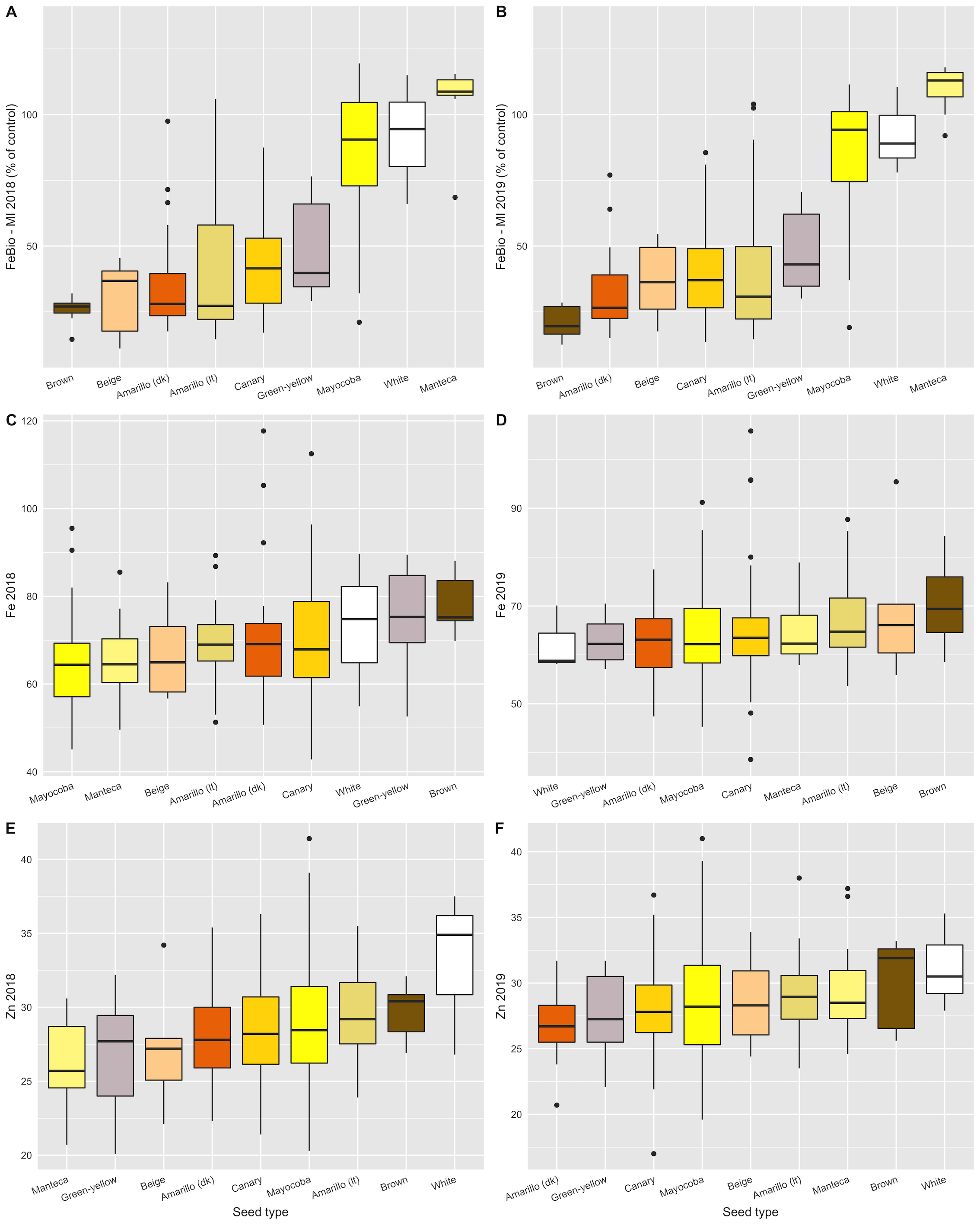

Supplement: Supplementary file 6 [file Image6.TIFF]

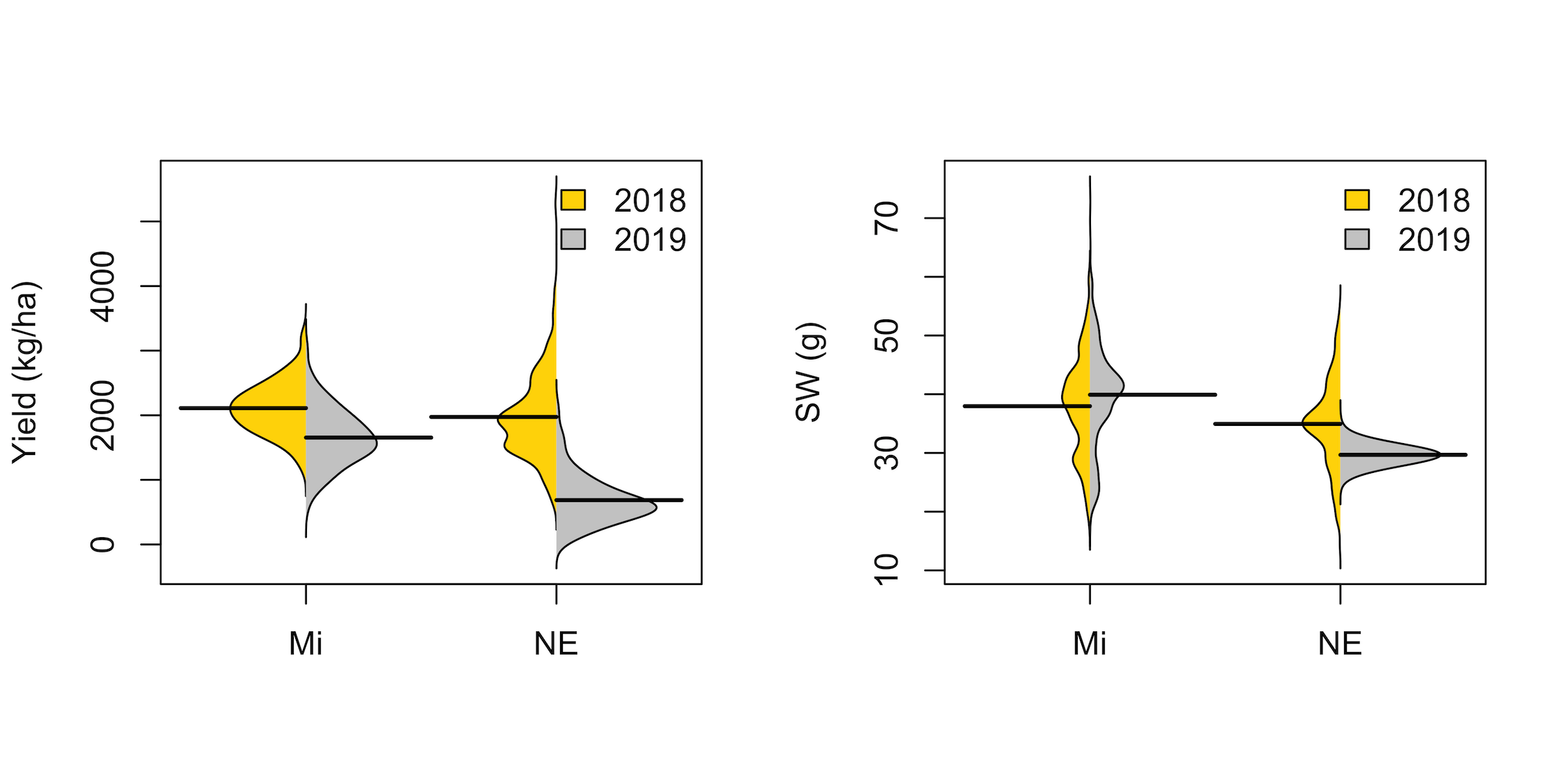

Supplement: Supplementary file 7 [file Image2.TIFF]

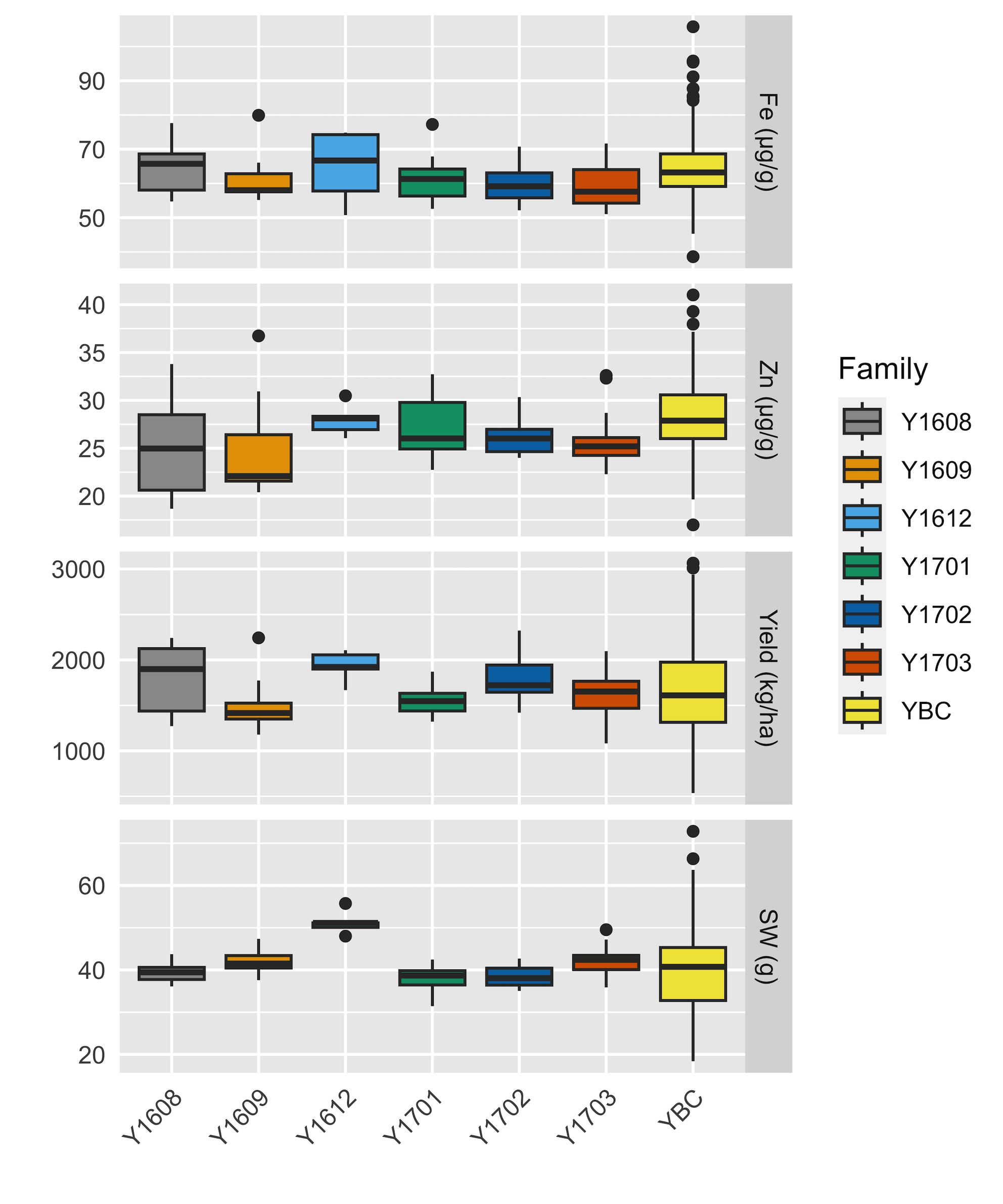

Supplement: Supplementary file 8 [file Image7.TIFF]
